# Prelamin A and Oct-1: a puzzle of aging

**DOI:** 10.18632/oncotarget.3422

**Published:** 2015-01-31

**Authors:** Arantza Infante, Clara I. Rodríguez

**Affiliations:** Stem Cells and Cell Therapy Laboratory, BioCruces Health Research Institute, Cruces University Hospital, Barakaldo, Spain

Aging is a tremendously complex biological process, in which several dys-regulated cellular pathways converge, such as alterations in the nuclear lamina which contribute to genomic instability, one of the hallmarks of aging [1]. The nuclear lamina is mainly composed of A and B-type lamins, type V intermediate filaments which establish a network that functions as a structural scaffold for the cell nucleus. Lamins interact also with chromatin and transcription factors functioning in DNA synthesis and gene transcription, therefore controlling indispensable cellular processes such as cell proliferation and differentiation. Prelamin A is the first unprocessed product of the *LMNA* gene which undergoes several posttranslational modifications before becoming mature lamin A, an essential component of nuclear lamina. Mutations in the *LMNA* gene or in genes that participate in lamin A maturation lead to the accumulation of lamin A precursors causing deep alterations in nuclear architecture, a hallmark of premature aging diseases such as Hutchinson-Gilford progeria syndrome or mandibuloacral dysplasia (reviewed in [2]). The finding that low levels of prelamin A are also present in cells of elderly individuals suggests that it exerts a role in physiological aging [3], reinforcing the relevance of premature aging cell models to study the molecular mechanisms underlying human aging.

It is worth noting that the mainly affected tissues (adipose tissue, bone, muscle) in *LMNA*-linked premature aging syndromes are from mesenchymal origin. In line with this finding, there is a decrease in the regenerative potential of adult stem cells in aging (reviewed in [1]). These observations suggest that the accumulation of prelamin A could be especially deleterious in human mesenchymal stem cells (hMSCs) in aging (physiological or premature). In fact, we demonstrated this hypothesis in a human experimental model based on prelamin A-accumulating hMSCs, which showed serious impediments to carry out a proper adipogenic differentiation [4].

In our latest work published in *Aging*, we focused on understanding the mechanisms underlying aging in a scenario of prelamin A accumulation, taking advantage of the experimental model mentioned above [5]. We initially confirmed that prelamin A-accumulating hMSCs exhibited telomere shortening and increased DNA damage response, both hallmarks of aging. Then, we found that prelamin A accumulation resulted in making hMSCs more sensitive to stress conditions, another characteristic of aged cells. Thus, increased ROS levels, impaired autophagy and alteration in the expression of genes related to aging, autophagy and oxidative stress only appeared in hMSCs exposed to a combination of prelamin A accumulation and stress conditions. Regarding autophagy, a cellular process which decays with age (reviewed in [1]), we found that autophagic flux was slowed down only under prelamin A accumulation and stress conditions, in spite of the inhibition of the mTORC1 pathway, a known repressor of autophagy. Importantly, the development of these aging characteristics resulted in a diminished functionality of prelamin A-accumulating hMSCs *in vivo*.

One possible pathogenic mechanism underlying prelamin A accumulation is that relevant levels of prelamin A could alter the network of the nuclear lamina, thus generating a trap for transcription factors. Confirming this hypothesis, we previously reported in our experimental model that prelamin A accumulation pathologically sequestered Sp1 transcription factor at the nuclear envelope of differentiated hMSCs [4]. We wondered then if the altered gene expression profile detected under prelamin A accumulation and stress could be attributed to the defective action of transcription factors. Bioinformatic analysis suggested that Oct-1, a transcription factor known to be associated with lamin B driving the oxidative stress response [6], could be governing this dys-regulated genetic program. We validated this *in-silico* result finding that accumulated prelamin A and stress conditions acted synergistically to enhance nuclear expression of Oct-1 and prelamin A itself. Furthermore, we found that Oct-1 activity under both these situations was inhibited in hMSCs. These results suggest that although overexpressed under stress signals, Oct-1 is not totally functional in hMSCs due to prelamin A accumulation. Moreover, we found that Oct-1 silencing resulted in an increase in senescence and autophagic flux in hMSCs.

Our findings reveal the relevance of this human experimental model to delve into the molecular mechanisms of human mesenchymal stem cell aging. Thus, we have found that the activity of Oct-1 can be attenuated by prelamin A accumulation in stressed hMSCs, and that this effect is, at least in part, responsible for the enhanced aging phenotype of these cells. Strengthening this observation, a later work published in *Aging* by the Latanzzi group showed that prelamin A sequestered Oct-1 at nuclear envelope in fibroblasts from premature aging patients [7]. These results clearly indicate that Oct-1 and prelamin A are two linked pieces in the complex puzzle of aging. Nevertheless, there are many questions deserving further studies, such as the intriguing observation that stress conditions seem to be a boost for the appearance/enhancement of some aging phenotypes in hMSCs that already accumulate prelamin A.
